# The clinical effect of Nano micelles containing curcumin as a therapeutic supplement in patients with COVID-19 and the immune responses balance changes following treatment: A structured summary of a study protocol for a randomised controlled trial

**DOI:** 10.1186/s13063-020-04824-y

**Published:** 2020-10-22

**Authors:** Mehdi Hassaniazad, Behnaz Rahnama Inchehsablagh, Hossein Kamali, Abdolali Tousi, Ebrahim Eftekhar, Mahmoud Reza Jaafari, Mohammad Fathalipour, Sara Nikoofal-Sahlabadi, Hamed Gouklani, Hesam Alizade, Amin Reza Nikpoor

**Affiliations:** 1 Infectious and Tropical Diseases Research Center, Hormozgan Health Institute, Hormozgan University of Medical Sciences, Bandar Abbas, Iran; 2 Student Research Committee, Faculty of Medicine, Hormozgan University of Medical Sciences, Bandar Abbas, Iran; 3 Department of Physiology, Faculty of Medicine, Hormozgan University of Medical Sciences, Bandar Abbas, Iran; 4 Endocrinology and Metabolism Research Center, Hormozgan University of Medical Sciences, Bandar Abbas, Iran; 5 Nanotechnology Research Center, Pharmaceutical Technology Institute, Mashhad University of Medical Sciences, Mashhad, Iran; 6 Department of Pharmaceutical Nanotechnology, School of Pharmacy, Mashhad University of Medical Sciences, Mashhad, Iran; 7 Biotechnology Research Center, Pharmaceutical Technology Institute, Mashhad University of Medical Sciences, Mashhad, Iran; 8 Department of Pharmacology and Toxicology, Faculty of Pharmacy, Hormozgan University of Medical Sciences, Bandar Abbas, Iran; 9 Department of Pharmaceutics, Faculty of Pharmacy, Hormozgan University of Medical Sciences, Bandar Abbas, Iran; 10 Molecular Medicine Research Center, Hormozgan Health Institute, Hormozgan University of Medical Sciences, Bandar Abbas, Iran

**Keywords:** COVID-19, Randomised controlled trial, protocol, Curcumin, Interleukins, Gene Expression, Lymphocytes

## Abstract

**Objectives:**

To investigates the effectiveness of curcumin-containing Nanomicelles as a therapeutic supplement in the treatment of patients with COVID-19 and its effect on immune responses balance changes following treatment.

**Trial design:**

This study is conducted as a prospective, placebo-controlled with parallel group, single-center randomized clinical trial on COVID-19 patients.

**Participants:**

Patients are selected from the COVID-19 ward of Shahid Mohammadi Hospital in Bandar Abbas, Iran.

Inclusion criteria:

1. Real time PCR-approved positive COVID-19 test.

2. Both gender

3. Age between 18 and 75 years

4. Signing a written consent

5. Lack of participation in other clinical trials

Exclusion criteria:

1. Pregnancy or lactation

2. Allergy to turmeric or curcumin

3. Smoking

4. Patient connected to the ventilator

5. SaO2 less than 90% or PaO2 less than 8 kPa

6. Having comorbidities (such as severe renal failure, Glomerular filtration rate less than 30 ml/min, liver failure, Congestive heart failure, or Chronic obstructive pulmonary disease)

7. History of gallstones

8. History of gastritis or active gastrointestinal ulcer

**Intervention and comparator:**

In addition to the routine standard treatments for COVID-19, in the intervention group, 40mg nanomicelles containing curcumin (SinaCurcumin Capsule, Exir Nano Sina Company, Iran), four times per day (after breakfast, lunch, dinner and before bedtime) and in the placebo group as the control group, capsules with the same appearance and characteristics (Placebo capsules, Exir Nano Sina Company, Iran) are prescribed for two weeks.

**Main outcomes:**

The effectiveness of Nano micelles containing curcumin treatment will be evaluated as daily clinical examinations of patients in both groups and, on days 0, 7 and 14, complete clinical symptoms and laboratory findings including peripheral blood and serum parameters such as inflammatory markers will be measured and recorded.

Moreover, in order to evaluate the balance of immune responses changes following treatments, serum level of IFN-γ, IL-17, Il-4 and TGF-β serum cytokines will be measured in both groups at time points of 0, 7 and 14 days post treatment. Gene expression of t-bet, GATA-3, FoxP3 and ROR- γT will also be measured at mentioned time points to assess the shift of T helper1, T helper2, T regulatory and T helper 17 immune responses following treatment.

**Randomisation:**

Randomized trials will be performed on 40 COVID-19 patients which will be randomized using encoded sealed boxes with computer generated random digits with 1:1 allocation ratio.

In order to randomization, placebo and SinaCurcumin Capsules will be numbered first by computer generated random digits. SinaCurcumin and placebo will then be stored and numbered in sealed packages based on generated random numbers. Finally, according to the order in which patients enter the study, packages are given to patients based on their number.

**Blinding (masking):**

The present study will be blind for all patients, physicians and nurses, laboratory technicians and statisticians.

**Numbers to be randomised (sample size):**

A total of 40 patients will be included in the study, 20 of them will be randomly assigned to the intervention group and 20 to the placebo group.

**Trial status:**

This is Version 1.0 of protocol dated 21 May 2020. The recruitment was started June 24, 2020 and is expected to be completed by October 31, 2020.

**Trial registration:**

This present clinical trial has been registered in the Iranian Registry of Clinical Trials (IRCT) with the registration code of “IRCT20200611047735N1”, https://www.irct.ir/trial/48843. Dated: 19 June 2020.

**Full protocol:**

The full protocol is attached as an additional file, accessible from the Trials website (Additional file 1). In the interest in expediting dissemination of this material, the familiar formatting has been eliminated; this Letter serves as a summary of the key elements of the full protocol.

**Supplementary information:**

**Supplementary information** accompanies this paper at 10.1186/s13063-020-04824-y.

## Supplementary information

**Additional file 1.** Full Study Protocol.

## Data Availability

Not applicable.

