# Supplementary material for: The clinical effect of Nano micelles containing curcumin as a therapeutic supplement in patients with COVID-19 and the immune responses balance changes following treatment: A structured summary of a study protocol for a randomised controlled trial
Source: Trials. 2020 Oct 22;21:876. doi: 10.1186/s13063-020-04824-y (PMC7578586; doi:10.1186/s13063-020-04824-y)
Supplement: Supplementary file 1 — Additional file 1. Full Study Protocol. [file 13063_2020_4824_MOESM1_ESM.docx]

**The clinical effect of Nano micelles containing curcumin as a therapeutic supplement in COVID-19 patients and investigating the immune responses balance changes following treatment: A structured summary of a study protocol for a randomised controlled trial**

Mehdi Hassani-Azad ^1^, Behnaz Rahnama Inchehsablagh ^2,3^, Hossein Kamali ^2^, Abdolali Tousi ^2^, Ebrahim Eftekhar ^4^, Mahmoud Reza Jaafari ^5,6,7^, Mohammad Fathalipour ^8,4^, Sara Nikoofal-Sahlabadi ^9^, Hamed Gouklani ^1^, Hesam Alizade ^1^, Amin Reza Nikpoor* ^10^

1. Infectious and Tropical Diseases Research Center, Hormozgan Health Institute, Hormozgan University of Medical Sciences, Bandar Abbas, Iran.

2. Student Research Committee, Faculty of Medicine, Hormozgan University of Medical Sciences, Bandar Abbas, Iran.

3. Department of Physiology, Faculty of Medicine, Hormozgan University of Medical Sciences, Bandar Abbas, Iran

4. Endocrinology and Metabolism Research Center, Hormozgan University of Medical Sciences, Bandar Abbas, Iran.

5. Nanotechnology Research Center, Pharmaceutical Technology Institute, Mashhad University of Medical Sciences, Mashhad, Iran.

6. Department of Pharmaceutical Nanotechnology, School of Pharmacy, Mashhad University of Medical Sciences, Mashhad, Iran

7. Biotechnology Research Center, Pharmaceutical Technology Institute, Mashhad University of Medical Sciences, Mashhad, Iran.

8. Department of Pharmacology and Toxicology, Faculty of Pharmacy, Hormozgan University of Medical Sciences, Bandar Abbas, Iran.

9. Department of Pharmaceutics, Faculty of Pharmacy, Hormozgan University of Medical Sciences, Bandar Abbas, Iran

10. Molecular Medicine Research Center, Hormozgan Health Institute, Hormozgan University of Medical Sciences, Bandar Abbas, Iran

Corresponding author:

Dr. Amin Reza Nikpoor. Ph.D.

Address: Molecular Medicine Research Center, Hormozgan Health Institute, Hormozgan University of Medical Sciences, Bandar Abbas, Iran.

Tel: (+98) 9177161727

Email: [nikpoora@hums.ac.ir](mailto:nikpoora@hums.ac.ir), [Nikpoora@gmail.com](mailto:Nikpoora@gmail.com).

**بررسی تاثیر نانومیسلهای حاوی کورکومین (سیناکورکومین) به عنوان مکمل درمانی در تسریع بهبودی بیماران مبتلا به کووید-19 و بررسی تغییرات بالانس پاسخ های ایمنی متعاقب درمان : یک کارآزمایی بالینی دو سوکور تصادفی کنترل شده با پلاسبو**

دانشگاه علوم پزشکی هرمزگان

کد اخلاق: HUMS.REC.1399.174

IRCT20200611047735N1

**مقدمه:**

علاوه بر خلاصه مشکل و ضرورت انجام پژوهش، لطفا با مرور متون به میزان نوآوری (باذکر کارهای مشابه در صورت وجود و ضرورت تکرار) و رابطه (احتمالی) با پیامدهای بیمار (مرگ و میر و ناتوانی) اشاره نمایید. همچنین هدف اصلی و سوال(های) اصلی پژوهش (سه تا حداکثر پنج سوال) در انتهای این بخش ذکر شود.

کورونا ویروسها با اندازه 160-80نانومتر، دارای ژنوم تک زنجیره مثبت RNA با طول 27 تا 34 کیلو باز هستند. ژنوم درون کپسید 20 وجهی بسته بندی شده است و در بیرونی ترین بخش ویروس، پوشش دو لایه فسفولیپیدی قرار گرفته است. ویروس این پوشش فسفولیپیدی را از غشا سلول میزبان بدست می آورد. سپس در داخل این غشا، گلیکو پروتئینهای ویروسی قرار میگیرند که یکی از وظایف آنها متصل کردن ویروس به گیرنده های سطح سلول میزبان است و بخش پروتئینی آن توسط خود ویروس رمز دهی شده و در سلول میزبان ساخته میشود. این ویروسها از طریق گلیکوپروتئین سطحی خود که در انولوپ قرار دارد به گیرنده ای در سطح سلولها بنام ACE2 ( enzyme converting-angiotensin-2) متصل میشوند که بیشتر در دیواره رگها بیان میشود .(1,2)

ویروس جدید کرونا یا 2 -CoV-SARS نام خود را از تلفیق سندروم شدید حاد تنفسی یا Acute Severe Syndrome Respiratory و خانواده این ویروس یعنی Coronaviride گرفته است. انتقال این ویروس از طریق قطرات تنفسی و همچنین تماس فرد سالم با فرد بیمار صورت میگیرد. دوره نهفتگی یا کمون بیماری 2 تا 14 روز است. علایم بیماری بیماران مبتلا به COVID-19 شامل تب، سرفه خشک و تنگی نفس می باشد. اسهال، تهوع/استفراغ نیز علایمی هستند که در برخی بیماران گزارش شده است. پنومونی یکی از علل اصلی مرگ و میر در مبتلایان میباشد (3,4).

بیماران مبتلا به COVID-19 ممکن است با پاسخ‌های ایمنی کنترل ‌نشده و متعاقب آن آسیب ریوی جدی و تهدیدکننده حیات مواجه شوند. بدن در پاسخ به این ویروس ممکن است به مقدار زیادی سلول ایمنی تولید کند که سایتوکاین‌های ترشح شده از آنها پدیده خطرناک طوفان سایتوکاینی را ایجاد می‌کنند. در طوفان سایتوکاینی، سایتوکاینهایی مانند IL-1, IL-6, IL-10, TNF-α آزاد می شوند. محققان این نظر را دارند که آزادسازی زیاد این سایتوکاینها در واکنش‌های شدید ایمنی و آسیب ریوی بیماران مبتلا به COVID-19 نقش دارد (5, 6, 7). در واقع طوفان سایتوکاینی یا cytokine release syndrome از علل اصلی مرگ و میر افراد مبتلا به کرونا به حساب میاد. به همین دلیل داروی مونوکلونال آنتی بادی توسیلیزومب که یک مهارکننده رسپتورIL-6 می باشد در درمان این طوفان سایتوکاینی کاربرد دارد و می تواند در درمان این بیماران کمک کند. کورکومین ترکیب اصلی گیاه زردچوبه می باشد. مطالعات برون تن و کارآزماییهای بالینی ثابت کرده است که کورکومین دارای اثرات آنتی اکسیدانی, ضد التهابی، ضد توموری و ایمونو مدولاتوری می باشد (8-14). علاوه بر این نشان داده شده است که کورکومین آزادسازی سایتوکاینهای زیادی رو مهار می کند که شامل موارد زیر می باشد: (15-24)

IL-1, IL-2, IL1β, IL6, IL8, Il-10- IL11, IL-12, IL-17, TNFα, interferon-γ, monocyte chemoattractant protein-1 (MCP1), macrophage inflammatory protein-1α (MIP1α), nuclear factor kappa-light-chain enhancer of activated B cells (NFĸB),

همچنین ساپرشن سایتوکاینهاتوسط کورکومین باعث بهتر شدن علایم بالینی در بیماریهای بوده است که در آنها طوفان سایتوکاینی دخیل می باشد. کورکومین در مدل موشی ویروسی سندرم دیسترس تنفسی حاد باعث کاهش سایتوکاینهایIL6, IL10, interferon-γ, MCP1 شده است و این کاهش سایتوکاینی باعث کاهش شدید التهاب و فیبروز در موشها بوده است (25). در مطالعه دیگر مصرف کورکومین در مدل موشی پانکراتیت حاد باعث ساپرشن میزان TNFα و صدمه به پانکراس شده است (26). مصرف کورکومین همچنین باعث ساپرشن سایتوکاینها به همراه بهبود علایم کلینیکی در مدلهای عفونتهای ویرال حاد شده است. مصرف کورکومین در موش باعث کاهش بیان IL1β, IL6, TNFα, NFĸB در موشهای عفونی شده با ویروس شده و موشها را در مقابل صدمه قلبی شدید ناشی از عفونت با این ویروس محافظت کرده است (27).

عامل اصلی ترشخ سیتوکاین ها در سیستم ایمنی، شبکه ای از سلول های ایمنی از جمله سلول های لنفوسیت T با مشخصه CD3, CD4 مثبت می باشند که T helper گفته می شود. سلول های T helper، دارای زیرکلاس های متفاوتی هستند که هر یک از ان ها با ترشخ سیتوکاین های مختلف، پاسخ های اصلی سیستم ایمنی را جهت دهی می نمایند. سلول های T helper 1 با مشخصه تولید , interferon-γ (IFN- γ) از اصلی ترین پاسخ های سیستم ایمنی بر علیه آنتی ژن های درون سلولی همانند ویروس ها و نیز عفونت های چرک زا می باشد که در نهایت تولید انتی بادی های IgG، فعال نمودن سلول های T cytotoxic و افزایش سلول کشی سلول های ماکروفاژی را ایجاد می نمایند. در سوی دیگر، سلول های T helper 2 با تولید سیتوکاین های IL-4, IL-5, IL-10 نقشی متضاد با سلول های T helper 1 را ایفا میکند که در بیماری کووید-19 می تواند نقش کاهش پاسخ های مخرب و افزایش بیش از حد پاسخ های T helper 1 را القا نماید. سلول های T regulatory از دیگر سلول های لنفوسیت T بوده که در تعدیل پاسخ های ایمنی نقش داشته و در بیماری کووید-19 می تواند در مراحل پاسخ های التهابی شدید را در ناحیه ریه، تعدیل نماید و مشخصه تولید سیتوکاین های این سلول شامل TGF-Bو IL-10 را می توان اشاره داشت. سلول های T helper17 نیز از دیگر سلول های T helper بوده که با تولید IL-17 می تواند در ایجاد ازدیاد التهاب در پاسخ به ویروس کووید-19 موثر باشد.

علاوه براین نشان داده شدهاست کورکومین فعالیت ضد ویروسی بر علیه ویروسهای مختلف زیر می باشد (28, 29):

human immunodeficiency virus-1 (HIV1), HIV2, herpes simplex virus (HSV), human papillomavirus (HPV), human T-lymphotropic virus-1 (HTLV1), hepatitis B virus (HBV), HCV, Japanese encephalitis virus

همچنین در مطالعات برون تن نشان داده شده است کورکومین فعالیت ضدویروسی بارزی بر علیه ویروسهای H1N1 دارد (30,31). از همه مهمتر این که نشان داده شده است کورکومین تولید پروتئینهای suppressor of cytokine signaling (SOCS) را تحریک می کند (32). این پروتینهای SOCS در محافظت کردن بر علیه طوفان سایتوکاینی شدید درموشهای عفونی شده با ویروس آنفلوانزا خیلی مهم می باشد (33).

فعالیت کورکومین در ساپرس کردن سایتوکاینهای متعدد و تاثیرش در مدلهای تجربی عفونهای ویروسی و بیماریهایی که همراه با طوفان سایتو کاینی می باشد پیشنهاد می کند که کورکومین در درمان بیماران مبتلا به COVID-19به عنوان ساپلمنت موثر باشد (34). ولی فراهمی زیستی خوراکی فرم معمول کورکومین به دلیل حلالیت در آب خیلی کم و متابولیسم سریع خیلی کم می باشد (35). ولی نانو.میسلهای حاوی کورکومین که به صورت کپسولهای سافت ژل خوراکی با نام تجاری سیناکورکومین در دسترس می باشند جذب افزایش یافته وتیمه عمر بالاتر در مقایسه با کورکومین معمول دارند (36). در این فرآورده تمام کورکومین در نانومیسلهای حدودا 10 نانومتری انکپسوله شده است و حلالیت در آب کورکومین بیش از 100000 برابر بیشتر شده است و فراهمی زیستی خوراکی آن خیلی بیشتر از فرم معمول کورکومین و فرآورده های تجاری موجود در بازار دنیا می باشد (حدودا 60 برابر)و تاثیر و بی ضرری و بی خطری آن در بیماریهای مختلف در کارآزماییهای بالینی به اثبات رسیده است (10, 36, 37, 38).

لازم به ذکر است که فرآورده سیناکورکومین از سال 1393 در بازار ایران موجود است و تا به حال بیش از 200000 بسته 50 تایی آن در دوزهای کپسول نرم 40 و 80 میلی گرمی به عنوان ساپلمنت برای درمان بیماریهای مختلف مصرف شده است و بی ضرری و بی خطری ان ثابت شده است. علاوه بر این این فرآورده برای بیش از 20 کارآزمایی بالینی ثبت شده برای درمان بیماریهای مختلف استفاده شده است که در این کارآزمایی های بالینی هم کارایی فرآورده برای درمان بیماریهای مختلف و هم عوارض جانبی ثبت شده است.

با توجه به مشکل طوفان سایتوکاینی ناشی از این ویروس و عدم درمان موثر یا واکسن برای این بیماری و همچنین اثربخشی و ویژگی های خاص نانومیسلهای حاوی کورکومین در کاهش قابل توجه سایتوکاین ها، مطالعه بالینی به منظور بررسی اثر بخشی نانومیسلهای حاوی کورکومین (سیناکورکومین) به عنوان ساپلمنت در درمان کورونا ویروس COVID-19)) انجام می گیرد و تغییر بالانس سلول های لنفوسیت T شامل سلول های T helper1، T helper 2، T regulatory و T helper 17 متعاقب مصرف نانومسیل های کورکومین در بیماران مبتلا به کووید -19 مورد مطالعه قرار خواهد گرفت.

**هدف اصلی:**

بررسی اثر بخشی نانومیسلهای حاوی کورکومین (سیناکورکومین) به عنوان درمان مکمل در بهبود / یا تخفیف علایم کلینیکی و یا بالینی بیماران مبتلا به کورونا ویروس COVID-19)) و بررسی تغییر بالانس سلول های T helper1، T helper 2، T regulatory و T helper 17 متعاقب درمان با نانومیسلهای حاوی کورکومین (سیناکورکومین).

**انجام طرح:**

در این مطالعه تعداد 40 بیمارکه طبق علایم کلینیکی و یافته های بالینی و آزمایشگاهی (جدول زیر) ثابت شده است مبتلا به کورونا ویروس COVID-19 می باشند و اندیکاسیون بستری را دارند ولی نیازمند انتوباسیون نیستند و شرایط ورود به مطالعه را تکمیل نموده اند وارد مطالعه شده و به طور تصادفی دوسویه ناآگاه در یکی از گروه های دارو یا دارونما قرار می گیرند. تمام بیماران طبق دستورالعمل درمانی COVID-19 درمان روتین را دریافت خواهند نمود. علاوه بر این در گروه دارو کپسول نانوکورکومین 40mg روز 4 عدد (بعد از صبحانه, ناهار و شام یکی وقبل از خواب یکی) به مدت 2 هفته و در گروه دارونما کپسول با ظاهر مشابه با همان شیوه و مدت تجویز می گردد. سپس سیر پیشرفت بیماری از نظر علایم کلینیکی و یافته های بالینی در گروهی که سیناکورکومین دریافت کرده اند با گروهی که پلاسبو دریافت کرده اند در زمانهای صفر، هفته 1، و هفته دوم مقایسه خواهند شد.

برای هربیمار یک CRF (Case Report Form) پر خواهد شد ودر انتهای مطالعه دو گروه با هم مقایسه خواهند شد. (فرم به پیوست ضمیمه شده است).


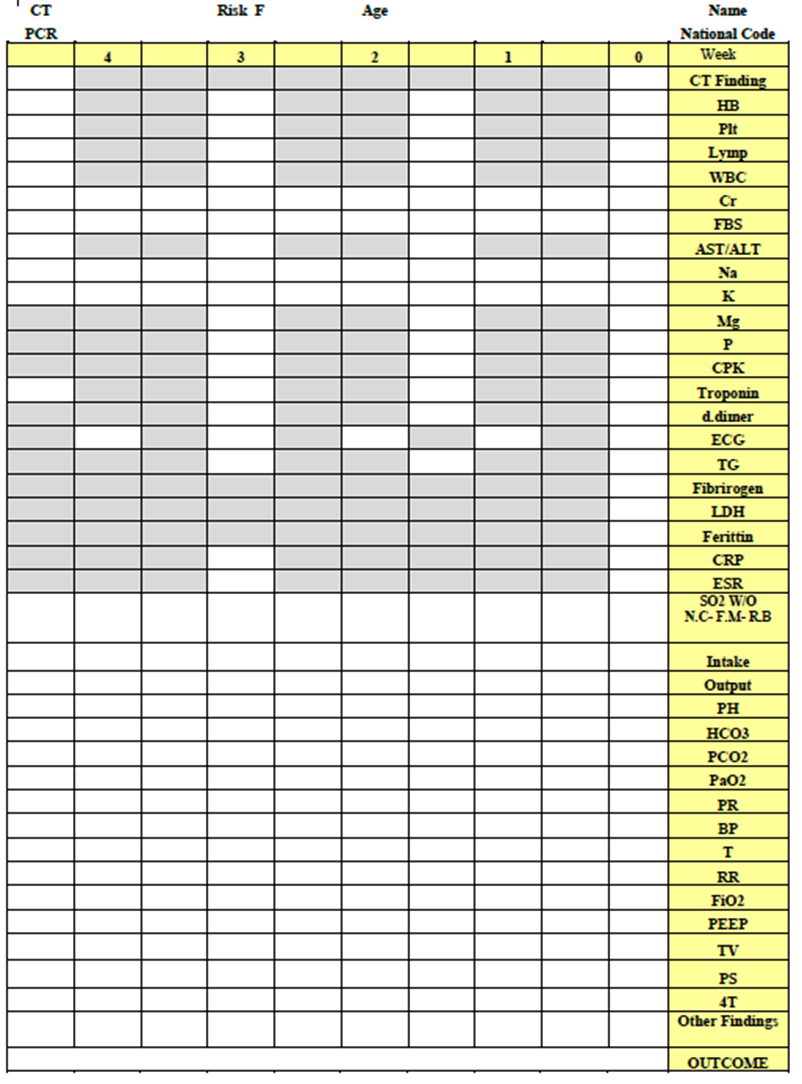


به علاوه، از بیماران در سه نوبت زمان های صفر، هفته اول و هفته دوم، خونگیری انجام شده و تست های ایمونولوژیک مربوط به بررسی بالانس پاسخ های ایمنی مورد بررسی قرار میگیرد. به این صورت که از بیماران 10 سی سی خون گرفته می شود.

به منظور بررسی تغییرات بالانس پاسخ های ایمنی در سلول های Helper، از بررسی میزان بیان ژن در سلول های خون محیطی و نیز سنجش سطح سرمی سیتوکاین ها استفاده می گردد.

به این منظور، پس از استخراج RNA و تولید cDNA، بیان ژن مربوط به فاکتورهای نسخه برداری اختصاصی سلول های ایمنی شامل: t-bet به منظور بررسی T helper 1، GATA-3 مربوط به بررسی T helper 2، Foxp3 به منظور بررسی T regulatory و نیز ROR- γT به منظور بررسی سلول های T helper -17 مورد بررسی قرا ر میگیرد. از ژن GAPDH نیز به عنوان ژن رفرانس مورد استفاده قرار میگیرد. در نهایت به منظور بررسی بیان ژن، از تنکنیک RT-PCR به شیوه Sybr green استفاده می گردد.

به این منظور، در سه نوبت خونگیری و استخراج RNA، می توان با بررسی میزان بیان ژن های مربوط به فاکتور های رونویسی اختصاصی هرکدام از زیرکلاس های لنفوسیت T، به میزان وجود ان ها در خون محیطی و تغییرات بالانس آن ها متعاقب درمان پی برد. از طرف دیگر، به این دلیل که عملکرد سلول های لنفوسیتی گفته شده در ایجاد شبکه های سیتوکاینی بوده و سیتوکاین ها نقش موثرو نیز مخربی را در پاسخ بدن به بیماری کووید19 ایفا می کنند، از افراد شرکت کننده در این مطالعه، در سه نوبت ذکر شده، خون گرفته و سرم آن جدا می گردد. بر روی سرم های جمع اوری شده، سیتوکاین هایγ IFN- به عنوان پاسخ اصلی سلول های T helper 1، سیتوکاین IL-4 به عنوان سیتوکاین اصلی پاسخ T helper 2، سیتوکاین TGF-B به عنوان پاسخ سلول های T regulatory و سیتوکاین IL-17 به عنوان پاسخ اصلی سلول های T helper 17 مورد سنجش سرمی به شیوه ELISA قرار خواهند گرفت.

**معیارهای ورود**

آزمایشات COVID-19 تایید شده آزمایشگاهی (2019-nCoV Real-Time RT-PCR) (ترجیحا در یک مرکز خاص و یا مراکز مورد تایید دانشگاه)صرف نظر از تظاهرات بالینی و سابقه تماس نزدیک

هردوجنس زن و مرد

سن بین 18 تا 75 سال

امضاء رضایت کتبی آگاهانه

عدم شرکت همزمان در سایر کارآزماییهای بالینی

**معیارهای خروج:**

بارداری و شیردهی

سابقه حساسیت به زردچوبه یا فرآرده کورکومین

استعمال دخانیات (بیش از 5 نخ سیگار در روز)

اتصال بیمار به ونتیلاتور

شواهد بالینی برای نارسایی تنفسی در زمان بستری/ پذیرش (SaO2 ≤ 90٪ یا PaO2 <8 kPa)

داشتن بیماریهای همراه (مانند نارسایی کلیوی شدیدGFR<30 ml/min، نارسایی کبد child C، CHF با EF<40% یا COPD)

سابقه سنگ کیسه صفرا

سابقه ورم معده یا زخم فعال گوارشی

**اهداف اختصاصی:**

1. بررسی اثرات درمانی نانومسیل های کورکومین(سیناکورکومین) در بهبود وضعیت بالینی بیماران کووید 19 در بین بیماران گروه درمان و پلاسبو
2. بررسی بیان فاکتور نسخه برداری اختصاصی t-bet سلول های لنفوسیتی T helper1 متعاقب درمان با نانومسیل های کورکومین(سیناکورکومین) در بین بیماران گروه درمان و پلاسبو در زمان های صفر، یک هفته و دوهفته پس از درمان
3. بررسی بیان فاکتور نسخه برداری اختصاصی GATA-3 سلول های لنفوسیتی T helper 2 متعاقب درمان با نانومسیل های کورکومین(سیناکورکومین) در بین بیماران گروه درمان و پلاسبو در زمان های صفر، یک هفته و دوهفته پس از درمان
4. بررسی بیان فاکتور نسخه برداری اختصاصی FoxP3 سلول های لنفوسیتی T regulatory متعاقب درمان با نانومسیل های کورکومین(سیناکورکومین) در بین بیماران گروه درمان و پلاسبو در زمان های صفر، یک هفته و دوهفته پس از درمان
5. بررسی بیان فاکتور نسخه برداری اختصاصی ROR- γT سلول های لنفوسیتی T helper 17 متعاقب درمان با نانومسیل های کورکومین(سیناکورکومین) در بین بیماران گروه درمان و پلاسبو در زمان های صفر، یک هفته و دوهفته پس از درمان
6. بررسی سطح سرمی سیتوکاین IFN-γ متعاقب درمان با نانومسیل های کورکومین(سیناکورکومین) در بین بیماران گروه درمان و پلاسبو در زمان های صفر، یک هفته و دوهفته پس از درمان
7. بررسی سطح سرمی سیتوکاین IL-4 متعاقب درمان با نانومسیل های کورکومین(سیناکورکومین) در بین بیماران گروه درمان و پلاسبو در زمان های صفر، یک هفته و دوهفته پس از درمان
8. بررسی سطح سرمی سیتوکاین TGF-B متعاقب درمان با نانومسیل های کورکومین(سیناکورکومین) در بین بیماران گروه درمان و پلاسبو در زمان های صفر، یک هفته و دوهفته پس از درمان
9. بررسی سطح سرمی سیتوکاین IL-17 متعاقب درمان با نانومسیل های کورکومین(سیناکورکومین) در بین بیماران گروه درمان و پلاسبو در زمان های صفر، یک هفته و دوهفته پس از درمان.

**سوالات پژوهش:**

1. آیا مصرف نانومسیل های کورکومین(سیناکورکومین) در بهبود وضعیت بالینی بیماران کووید 19 در بین بیماران گروه درمان و پلاسبو تاثیر دارد؟
2. بیان فاکتور نسخه برداری اختصاصی t-bet سلول های لنفوسیتی T helper1 متعاقب درمان با نانومسیل های کورکومین(سیناکورکومین) در بین بیماران گروه درمان و پلاسبو در زمان های صفر، یک هفته و دوهفته پس از درمان به چه صورت می باشد؟
3. بیان فاکتور نسخه برداری اختصاصی GATA-3 سلول های لنفوسیتی T helper 2 متعاقب درمان با نانومسیل های کورکومین(سیناکورکومین) در بین بیماران گروه درمان و پلاسبو در زمان های صفر، یک هفته و دوهفته پس از درمان به چه صورت می باشد؟
4. بیان فاکتور نسخه برداری اختصاصی FoxP3 سلول های لنفوسیتی T regulatory متعاقب درمان با نانومسیل های کورکومین(سیناکورکومین) در بین بیماران گروه درمان و پلاسبو در زمان های صفر، یک هفته و دوهفته پس از درمان به چه صورت می باشد؟
5. بیان فاکتور نسخه برداری اختصاصی ROR- γT سلول های لنفوسیتی T helper 17 متعاقب درمان با نانومسیل های کورکومین(سیناکورکومین) در بین بیماران گروه درمان و پلاسبو در زمان های صفر، یک هفته و دوهفته پس از درمان به چه صورت می باشد؟
6. سطح سرمی سیتوکاین IFN-γ متعاقب درمان با نانومسیل های کورکومین(سیناکورکومین) در بین بیماران گروه درمان و پلاسبو در زمان های صفر، یک هفته و دوهفته پس از درمان به چه صورت می باشد؟
7. سطح سرمی سیتوکاین IL-4 متعاقب درمان با نانومسیل های کورکومین(سیناکورکومین) در بین بیماران گروه درمان و پلاسبو در زمان های صفر، یک هفته و دوهفته پس از درمان به چه صورت می باشد؟
8. سطح سرمی سیتوکاین TGF-B متعاقب درمان با نانومسیل های کورکومین(سیناکورکومین) در بین بیماران گروه درمان و پلاسبو در زمان های صفر، یک هفته و دوهفته پس از درمان به چه صورت می باشد؟
9. سطح سرمی سیتوکاین IL-17 متعاقب درمان با نانومسیل های کورکومین(سیناکورکومین) در بین بیماران گروه درمان و پلاسبو در زمان های صفر، یک هفته و دوهفته پس از درمان به چه صورت می باشد؟

1. Ziebuhr J. Advances in Virus Research. Coronaviruses, Volume 96.1st Edition, 2016; Hardcover ISBN: 9780128047361.

2. Corman VM, Muth D, Niemeyer D, Drosten C (2018). "Hosts and Sources of Endemic Human Coronaviruses". Advances in Virus Research. 100: 163– 188. doi:10.1016/bs.aivir.2018.01.001.

3. Gorbalenya AE (11 February 2020). "Severe acute respiratory syndrome-related coronavirus – The species and its viruses, a statement of the Coronavirus Study Group". bioRxiv. doi:10.1101/2020.02.07.937862.

4. El Sahly HM. "Genomic Characterization of the 2019 Novel Coronavirus". New England Journal of Medicine. Retrieved 9 February 2020.

5. Wauquier N, Becquart P, Padilla C, Baize S and Leroy EM: Human fatal Zaire Ebola virus infection is associated with an aberrant innate immunity and with massive lymphocyte apoptosis. PloS Negl Trop Dis 4(10) http://dx.doi.org/ 10.1371/journal.pntd. 2010.0000837.

6. Villinger F, Rollin PE, Brar SS, Chikkala NF, Winter J, Sundstrom JB, Zaki SR, Swanepoel R, Ansari AA and Peters CJ (1999), Markedly elevated levels of interferon (IFN)γ, IFNα, interleukin (IL)-2, IL10 and tumor necrosis factor-α associated with fatal Ebola virus infection. J Infect Dis 179: S188-S191.

7. Teijaro JR, Walsh KB, Rice S, Rosen H and Oldstone MBA (2014), Mapping the innate signaling cascade essential for cytokine storm during influenza virus infection. Proc Natl Acad Sci 111: 3799-3804.

8. Prasad, S., et al., (2014) Curcumin, a component of golden spice: from bedside to bench and back. Biotechnol Adv, **32**(6): 1053-64.

9. Panahi, Y., et al., (2012) A randomized controlled trial on the anti-inflammatory effects of curcumin in patients with chronic sulphur mustard-induced cutaneous complications. Ann Clin Biochem, **49**(Pt 6): 580-8.

10. Hashemzadeh K, Davoudian N, Jaafari MR, Mirfeizi Z. (2019), [The Effect of Nanocurcumin on the Improvement Symptoms of Knee Osteoarthritis: A Randomized Clinical Trial.](https://www.ncbi.nlm.nih.gov/pubmed/31868149) Curr Rheumatol Rev. doi: 10.2174/1874471013666191223152658.

11. Panahi, Y., et al., (2015) Antioxidant and anti-inflammatory effects of curcuminoid-piperine combination in subjects with metabolic syndrome: A randomized controlled trial and an updated meta-analysis. Clin Nutr, **34**(6): 1101-8.

12. Panahi, Y., et al., (2014), Curcuminoid treatment for knee osteoarthritis: a randomized double-blind placebo-controlled trial. Phytother Res, **28**(11): 1625-31.

13. Sahebkar, A., et al., (2016), Curcumin downregulates human tumor necrosis factor-alpha levels: A systematic review and meta-analysis ofrandomized controlled trials. Pharmacol Res, **107**: 234-42.

14. Yousefi F, Lavi Arab F, Jaafari MR, Rastin M, Tabasi N, Hatamipour M, Nikkhah K, Mahmoudi M. (2019), [Immunoregulatory, proliferative and anti-oxidant effects of nanocurcuminoids on adipose-derived mesenchymal stem cells.](https://www.ncbi.nlm.nih.gov/pubmed/31338010) EXCLI J.;18:405-421. doi: 10.17179/excli2019-1366

15. Abe Y, Hashimoto S and Horie T (1999), Curcumin inhibition of inflammatory cytokine production by human peripheral blood monocytes and alveolar macrophages. Pharmacol Res 39: 41-47.

16. Jain SK, Rains J, Croad J, Larson B and Jones K (2009), Curcumin supplementation lowers TNFα, IL6, IL8, and MCP1 secretion in high glucose-treated cultured monocytes and blood levels of TNFα, IL6, MCP1, glucose, and glycosylated hemoglobin in diabetic rats. Antioxid Redox Signal 11: 241–249.

17. Kloesch B, Becker T, Dietersdorfer E, Kiener H and Steiner G (2013), Anti-inflammatory and apoptotic effects of the polyphenol curcumin on human fibroblast-like synoviocytes. Int Immunopharmacol 15: 400-405.

18. Raflee P, Nelson VM, Manley S, Wellner M, Floer M, Binion DG and Shaker R (2009). Effect of curcumin on acidic pH-induced expression of IL6 and IL8 in human esophageal epithelial cells (HET1A): Role of PKC, MAPKs, and NFĸB. Amer J PhysiolGastrointest Liver Physiol 296: G388-G398.

19. Biswas SK, McClure D, Jimenez LA, Megson IL and Rahman I (2005), Curcumin induces glutathione biosynthesis and inhibits NFĸB activation and interleukin-8 release in alveolar epithelial cells: Mechanism of free radical scavenging activity. Antioxid Redox Signal 7: 32-41.

20. Xu YX, Pindolia KR, Janakiraman N, Chapman RA and Gautam SC (1997), Curcumin inhibits IL1α and TNFα induction of AP1 and NFĸB DNA-binding activity in bone marrow stromal cells. Hematopathol Mol Hematol 11: 49-62.

21. Jobin C, Bradham CA, Russo MP, Juma B, Narula AS, Brenner DA and Sartor RB (1999), Curcumin blocks cytokine-mediated NFĸβ activation and proinflammatory gene expression by inhibiting inhibitory factor Iĸβ kinase activity. J Immunol 163: 3474-3483.

22. Henrotin Y, Clutterbuck AL, Allaway D, Lodwig EM, Harris P, Mathy-Hartert M, Shakibaei M and Mobasheri A (2010), Biological actions of curcumin on articular chondrocytes. Osteoarthr Cartil 18: 141-149.

23. Gao X, Kuo J, Jiang H, Deeb D, Liu Y, Divine G, Chapman RA, Dulchavsky SA and Gautam SC (2004), Immunomodulatory activity of curcumin: Suppression of lymphocyte proliferation, development of cell-mediated cytotoxity, and cytokine production in vitro. Biochem Pharmacol 68: 51-61.

24. Fahey AJ, Robins RA and Constantinescu CS (2007), Curcumin modulation of IFNβ and IL12 signaling and cytokine induction in human T-cells. J Cell Mol Med 11: 1129-1137.

25. Avasarala S, Zhang F, Liu G, Wang R, London SD and London L (2013) Curcumin modulates the inflammatory response and inhibits subsequent fibrosis in a mouse model of viral-induced acute respiratory distress syndrome. PLoS ONE 8(2) http:// dx.doi.org/10.1371/journal.pone. 2013.0057285.

26. Yu WG, Xu G, Ren GJ, Xu X, Yuan HQ, Qi XL and Tian KL (2011) Preventive action of curcumin in experimental acute pancreatitis in mouse. Indian J Med Res 134: 717-724.

27. Song Y, Ge W, Cai H and Zwang H (2013), Curcumin protects mice from coxsackie virus B3-induced myocarditis by inhibiting the phosphatidylinositol 3 kinase/Akt/nuclear factor-ĸB pathway. J Cardiovasc Pharmacol Ther 18: 560-569.

28. Moghadamtousi SZ, Kadir HA, Hassandarvish P, Tajik H, Abubakar S and Zandi K (2014), A review on antibacterial, antiviral, and antifungal activity of curcumin. Biomed Res Int <http://dx.doi.org/10.1155/2014.186864>.

29. Mathew D, Hsu WL (2018) Antiviral potential of curcumin, Journal of Functional Foods 40 692–699.

30. Chen DY, Shien JH, Tiley L, Chiou SS, Wang SY, Chang TJ, Lee YJ, Chan KW and Hsu WL (2010), Curcumin inhibits influenza virus infection and haemagglutination activity. Food Chem 119: 1346- 1351.

31. Ou JL, Mizushina Y, Wang SY, Chuang DY, Nadar M and Hsu WL (2013), Structure–activity relationship analysis of curcumin analogues on anti-influenza virus activity. FEBS J 280: 5829- 5840.

32. Chen CQ, Yu K, Yan QX, Xing CY, Chen Y, Yan Z, Shi YF, Zhao KW and Gao SM (2013), Pure curcumin increases the expression of SOCS1 and SOCS3 in myeloproliferative neoplasms through suppressing class I histone deacetylases. Carcinogenesis 34: 1442-1449.

33. Kedzierski L, Linossi EM, Kolesnik TB, Day EB, Bird NL, Kile BT, Belz GT, Metcalf D, Nicola NA, Kedzierska K and Nicholson SE (2014), Suppressor of cytokine signaling 4 (SOCS4) protects against severe cytokine storm and enhances viral clearance during influenza infection. PLoS Pathog 10 (5) http://dx.doi.org/10:1371/journal.ppat.2014.1004134.

34. Sordillo PP, Helson L, Curcumin Suppression of Cytokine Release and Cytokine Storm. (2015) A Potential Therapy for Patients with Ebola and Other Severe Viral Infections. in vivo 29: 1-4.

35. Liu, W., et al., (2016) Oral bioavailability of curcumin: problems and advancements. J Drug Target, **24** (8): 694-702.

36. Hatamipour M, Sahebkar A, Alavizadeh SH, Dorri M, Jaafari MR. 2019 Mar; [Novel nanomicelle formulation to enhance bioavailability and stability of curcuminoids.](https://www.ncbi.nlm.nih.gov/pubmed/31156789) Iran J Basic Med Sci22(3):282-289. doi: 10.22038/ijbms.2019.32873.7852.

37. Ahmadi M, Agah E, Nafissi S, Jaafari MR, Harirchian MH, Sarraf P, Faghihi-Kashani S, Hosseini SJ, Ghoreishi A, Aghamollaii V, Hosseini M, Tafakhori A. 2018 April, [Safety and Efficacy of Nanocurcumin as Add-On Therapy to Riluzole in Patients With Amyotrophic Lateral Sclerosis: A Pilot Randomized Clinical Trial.](https://www.ncbi.nlm.nih.gov/pubmed/29352425) Neurotherapeutics. 15 (2): 430-438. doi: 10.1007/s13311-018-0606-7.

38. Jazayeri-Tehrani SA, Rezayat SM, Mansouri S, Qorbani M, Alavian SM, Daneshi-Maskooni M, Hosseinzadeh-Attar MJ (2018), The nanocurcumin reduces appetite in obese patients with nonalcoholic fatty liver disease (nafld): a double-blind randomized placebo-controlled clinical trial. Nanomed. J. 5(2): 67-76.
